# Cardiology Assessment of Patients Undergoing Evaluation for Orthotopic Liver Transplantation

**DOI:** 10.1016/j.jscai.2022.100528

**Published:** 2022-11-25

**Authors:** Michael S. Lee, Subeer Wadia, Yerem Yeghiazarians, Ray Matthews, Christopher J. White, Howard C. Herrmann, William O’Donnell, John McPherson, Massoud A. Leesar, Rolf P. Kreutz, Danielle Brandman, Anuj Gupta, Stacy Mandras, David E. Kandzari

**Affiliations:** a Division of Cardiology, University of California, Los Angeles Medical Center, Los Angeles, California; b Division of Cardiology, University of California, San Francisco Medical Center, San Francisco, California; c Division of Cardiology, University of Southern California Medical Center, Los Angeles, California; d Division of Cardiology, Ochsner Medical Center, New Orleans, Louisiana; e Division of Cardiology, University of Pennsylvania Medical Center, Philadelphia, Pennsylvania; f Division of Cardiology, Vanderbilt University Medical Center, Nashville, Tennessee; g Division of Cardiology, University of Alabama at Birmingham Medical Center, Birmingham, Alabama; h Division of Cardiovascular Medicine, Indiana University Health/Indiana University School of Medicine, Indianapolis, Indiana; i Division of Hepatology, University of California, San Francisco Medical Center, San Francisco, California; j Division of Cardiology, University of Maryland, Baltimore, Maryland; k Division of Cardiology, Advent Health, Orlando, Florida; l Division of Cardiology Piedmont Heart Institute, Atlanta, Georgia

**Keywords:** coronary angiography, pulmonary hypertension, structural heart disease

## Abstract

Orthotopic liver transplantation (OLT) is a viable treatment option for end-stage liver disease. Significant perioperative stress is placed on the cardiovascular system because of hemodynamic changes and the length of the operation. Diagnosis and treatment of cardiovascular disease before OLT are imperative to ensure favorable outcomes. Considerable variability exists among practitioners caring for these patients. Institutions tailor their protocols on the basis of local and historical practices, the preferences of the cardiologists, and the OLT team, and algorithms are not often revised or updated on the basis of the available evidence. In collaboration with cardiology and hepatology experts from leading OLT centers, we sought to examine the diagnostic cardiovascular workup of OLT candidates, including a review of the available literature on the diagnostic modalities used to screen cardiovascular disease before OLT. We advocate an emphasis on noninvasive methods to assess cardiovascular risk with reserved use of invasive risk stratification in select patients.

## Highlights

• Liver transplantation is a viable treatment option for end-stage liver disease.
• Algorithms are not updated based on the available evidence.
• Noninvasive methods to assess cardiovascular risk is preferred.

## Graphical abstract

**Figure:**
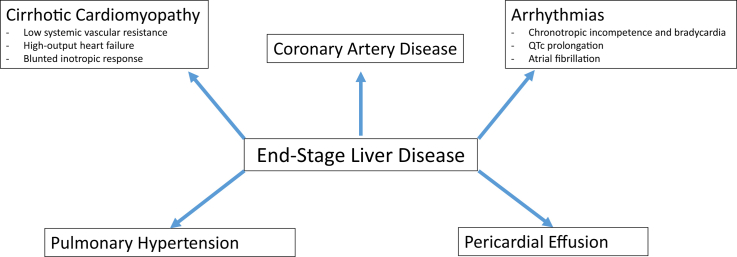

## Introduction

Orthotopic liver transplantation (OLT) is a high-risk treatment option for patients who have end-stage liver disease (ESLD).^1^ The United Network for Organ Sharing reported that 8895 OLTs were performed in the United States in 2019, representing a 7.8% increase from 2018 (8250 OLTs).^2^ Many OLT candidates are older and have comorbidities, including cardiovascular disease, which is a common cause of morbidity and mortality following OLT.^3^ In a multivariate analysis, the risk of death after OLT was most strongly associated with a history of coronary artery disease (CAD), angiographically confirmed coronary stenosis, and a left ventricular ejection fraction (LVEF) below 50%.^4^^,^^5^

The primary objective of a cardiology consultation for OLT candidates is to provide cost-effective risk stratification and guide appropriate treatment. A state-of-the-art review published almost a decade ago recommended that OLT candidates undergo rest and stress echocardiography followed by invasive catheterization for appropriately selected patients depending on their risk factors.^6^^,^^7^ However, preoperative cardiovascular evaluation and treatment vary widely among institutions, which tailor their protocols based on the preferences of individual cardiologists and historical and anecdotal practices rather than robust clinical trial data. Furthermore, these protocols are not often revised or updated on the basis of evolving practices and evidence basis. Institutional protocols may also be influenced by the OLT team (transplant surgeons, hepatologists, and anesthesiologists), which has a more limited experience with the diagnosis and management of cardiovascular disease. The purpose of this consensus document is to review current institutional practices and literature to provide opinions and recommendations regarding the preoperative cardiology assessment of OLT candidates made by authors who have specific interest and expertise in this subject area.

## Methodology

The authors of this document are cardiologists and hepatologists from large OLT centers in the United States with a broad range of expertise in the diagnostic cardiovascular workup of OLT candidates. Authors corresponded to share their institutional practices to generate a consensus document for the evaluation and management of OLT candidates. A general framework by the authors was used to conduct a comprehensive literature review to summarize existing evidence, indicate gaps in current knowledge, and formulate recommendations. Only English-language studies were reviewed, with PubMed/MEDLINE as the primary resource. The conclusions of this document reflect the views and opinions of the authors. All authors had the opportunity to comment on the initial drafts and approved the final version of this document.

## Perioperative considerations

Orthotopic liver transplantation requires a complex operation involving vascular reconstruction of the hepatic artery, portal vein, and hepatic venous drainage into the inferior vena cava.^8^^,^^9^ Significant perioperative stress is imposed on the cardiovascular system because of hemodynamic perturbations and surgical duration (up to 12 hours). The mechanisms of increased perioperative cardiovascular demand may include massive bleeding and transfusion requirements, inferior vena cava clamping, hypotension, and postreperfusion syndrome with a sudden increase in preload contributing to a rise in central venous and pulmonary arterial pressures.

## Pathophysiology in patients with ESLD

Patients with ESLD may manifest deleterious pathophysiologic changes that may pose cardiovascular risk during OLT. These include bradycardia and low systemic vascular resistance because of relaxed vasomotor tone, leading to hypotension (Central Illustration). In addition, left ventricular hypertrophy is more commonly observed in patients with ESLD,^10^ and increased ventricular stiffness and impaired myocardial relaxation can lead to diastolic dysfunction.^11^ Left ventricular hypertrophy, decreased preload, and low systemic vascular resistance may also lead to elevated cardiac output. Hemodynamically significant left ventricular outflow tract obstruction can be due to left ventricular hypertrophy and a hyperdynamic left ventricle. Cardiac output is commonly elevated, leading to a high output state, and may be exacerbated by intrahepatic arteriovenous fistulae in chronic liver disease. These patients may demonstrate an entity known as “cirrhotic cardiomyopathy,” which is characterized by attenuated systolic and diastolic function, electrophysiologic changes such as prolonged repolarization and chronotropic incompetence, and blunted cardiac output response to beta-adrenergic stimulation.^12^^,^^13^ The severity of the cardiomyopathy may remain clinically inapparent and manifest only at the time of OLT. Anesthesia, mechanical ventilation, and fluid management may place added strain and demand on an already exhausted cardiac reserve, resulting in perioperative hypervolemia and pulmonary edema. Postoperative pulmonary edema is estimated to affect nearly 6% of all OLT recipients, and severe heart failure after OLT is associated with worse outcomes.^14^, ^15^, ^16^

**Central Illustration:**
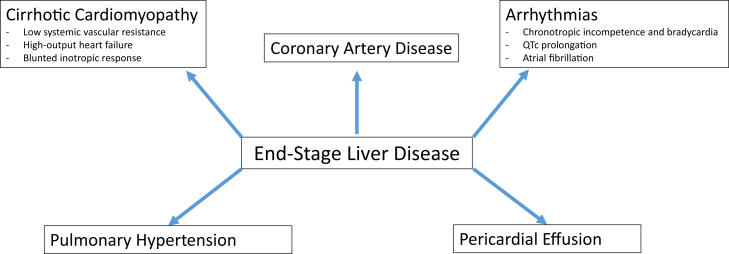
Pathophysiology in patients with end-stage liver disease. QTc, corrected QT.

## Perioperative evaluation

### Electrocardiography

**Figure 1:**
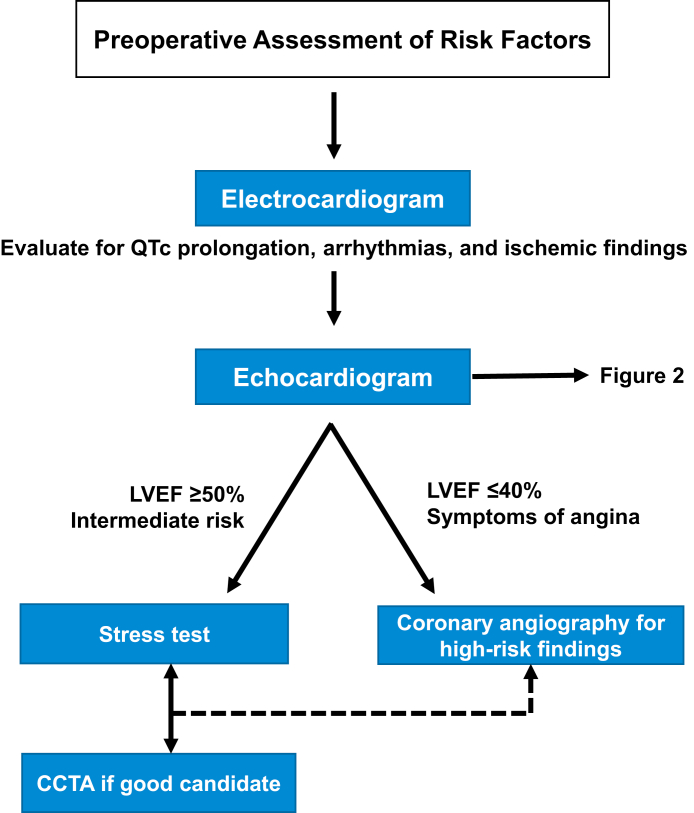
**Preoperative assessment.** CCTA, coronary computed tomography angiography; LVEF, left ventricular ejection fraction; QTc, corrected QT.

An electrocardiogram should be obtained in all OLT candidates to identify disturbances in heart rhythm, conduction abnormalities, and abnormalities suggestive of myocardial ischemia (Figure 1). The presence of corrected QT (QTc) prolongation, defined as QTc of >0.47 seconds in women and >0.45 seconds in men, should prompt an evaluation for electrolyte abnormalities that may require treatment, with particular attention to the maintenance of serum potassium level of ≥4 mmol/L and magnesium level of ≥2 mmol/L. The medication list should be reviewed to identify any agent that may promote QTc prolongation. Tacrolimus, which is an immunosuppressant commonly used after OLT, can prolong the QTc interval. Therefore, the optimization of electrolytes before OLT and surveillance after OLT is required to minimize the risk of torsades de pointes.

### Echocardiography

Transthoracic echocardiography (TTE) should also be obtained in all OLT candidates because it remains the mainstay imaging modality to assess biventricular and valvular function, estimate pulmonary artery systolic pressure (PASP), and identify the presence of pericardial effusion.

#### Ventricular function

Controversy surrounds the minimum LVEF that would be acceptable for OLT. Patients with LVEF of <50% are at risk of perioperative complications and should be carefully evaluated using a multidisciplinary approach that includes the cardiologist and OLT team, taking into account symptoms and functional class. Consistent with the 2012 appropriate use criteria for diagnostic catheterization, patients with left ventricular systolic dysfunction (LVEF of <40%) or segmental wall motion abnormalities should be referred for left heart catheterization and coronary angiography for hemodynamic evaluation and to exclude ischemic cardiomyopathy.^17^ Repeat TTE should be performed after guideline-directed medical therapy, including cardiac resynchronization therapy, if appropriate. Although data are limited, combined heart-liver transplantation can be considered in select patients. Tricuspid annular plane systolic excursion can be used to assess right ventricular function. Decreased right ventricular function increases the risk of perioperative complications.

#### Valvular function

Accurate assessment of the severity of valvular lesions in patients with advanced liver failure can be challenging because of the high output state, increasing the flow and gradients across such lesions. Stenotic lesions, particularly significant mitral or aortic stenosis, may be worsened by the high flow physiologic state in patients with ESLD and poorly tolerated in the peritransplant period. Because of the high risk posed by cardiopulmonary bypass in patients with ESLD, less invasive therapies are preferred. Transcatheter aortic valve replacement is a reasonable treatment option for aortic stenosis in patients with ESLD awaiting OLT who have high surgical morbidity and mortality but who otherwise are good candidates for OLT with life expectancy (including with OLT) of >1 year.^18^ Mitral and tricuspid regurgitation and pulmonary hypertension that is the result of volume overload can improve after volume optimization. Although data are lacking in patients with ESLD, the MitraClip (Abbott) procedure is a minimally invasive catheter-based therapy that can be considered for treating moderate-to-severe mitral regurgitation.

#### Pulmonary artery pressure

**Figure 2:**
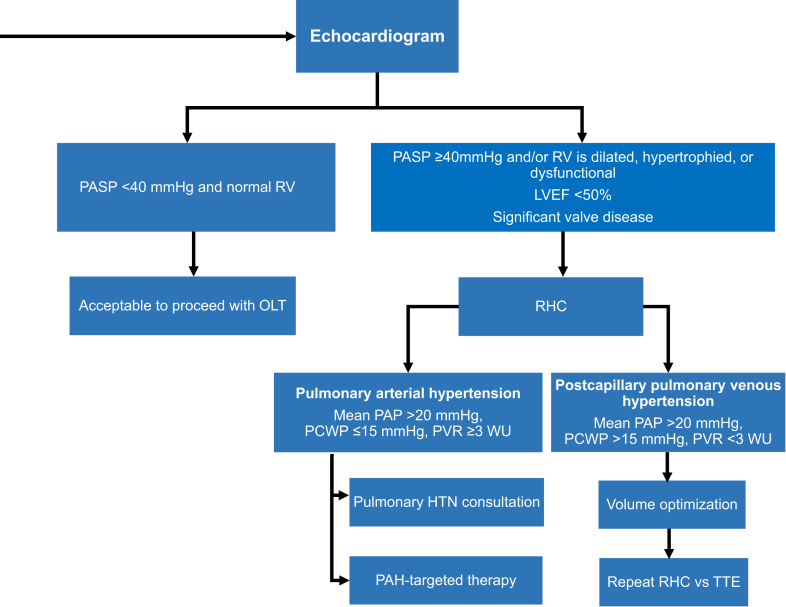
**Echocardiogram.** HTN, hypertension; LVEF, left ventricular ejection fraction; OLT, orthotopic liver transplantation; PAP, pulmonary artery pressure; PASP, pulmonary artery systolic pressure; PCWP, pulmonary capillary wedge pressure; PVR, pulmonary vascular resistance; RHC, right heart catheterization; RV, right ventricle; TTE, transthoracic echocardiogram; WU, Wood units.

The PASP is commonly elevated in patients with ESLD. The injection of agitated saline during TTE can augment the tricuspid regurgitation jet if it is not well visualized. An acceptable PASP for patients undergoing OLT is <40 mm Hg when the right ventricle is structurally normal (Figure 2). Right heart catheterization (RHC) is recommended if the PASP is ≥40 mm Hg and/or the right ventricle is dilated, hypertrophied, or dysfunctional. Given the significant fluid shifts during OLT and increased volume load on the heart after OLT, RHC is also recommended if the LVEF is <50% or in the presence of concomitant valve disease. Although the International Liver Transplantation Society recommends mandatory screening for portopulmonary hypertension (PoPH) with TTE in OLT candidates, the optimal cutoff for estimated PASP has not been universally decided. In an analysis of cutoff values, a PASP of 38 mm Hg yields a maximal specificity of 82% and a sensitivity and negative predictive value of 100% for PoPH.^19^ The results from a prospective study examining the relationship between screening TTE and RHC suggest that a right ventricular systolic pressure threshold of 50 mm Hg on TTE predicts moderate-to-severe PoPH with a positive and negative predictive value of 74% and 97%, respectively.^20^ As such, all patients with elevated PASP on TTE should proceed with RHC for confirmation. The lack of an adequate tricuspid regurgitation jet precluding the accurate measurement of the PASP does not necessarily warrant RHC unless the right ventricle is structurally abnormal or there are other echocardiographic signs of pulmonary hypertension.

#### Patent foramen ovale

Patent foramen ovale (PFO) represents an interatrial communication found incidentally in 25% to 30% of the general population. The presence of early microbubbles (<4 cardiac cycles) from agitated saline injection during TTE can be used to diagnose a PFO. Intracardiac shunting from a PFO should be distinguished from other etiologies of positive bubble study, such as intrapulmonary shunting or hepatopulmonary syndrome, which has a higher incidence in patients with ESLD (ranging from 5% to 47% in the literature).^21^ The increased preload from reperfusion following OLT may predispose to a change in flow dynamics across the PFO. Furthermore, there is concern about air or thrombotic emboli, which could increase the risk of perioperative cerebrovascular events.^22^ No controlled studies have demonstrated a benefit of prophylactic PFO closure before OLT, and observational studies have demonstrated mixed results.^23^ Given insufficient data, PFO closure before OLT is not universally recommended, should be guided by a multidisciplinary approach on an individualized basis, and should be reserved for those who have appropriate indications unrelated to OLT candidacy.

#### Pericardial diseases and systemic conditions

Pericardial effusion occurs in up to 22% of patients with ESLD, likely resulting from low oncotic pressures associated with decompensated cirrhosis, resulting in edema and fluid retention.^24^ Significant pericardial effusion is uncommon in patients with ESLD. Large pericardial effusion with hemodynamic significance should be evaluated for pericardiocentesis. A pericardial window should be performed if pericardiocentesis is not feasible or if there is recurrent effusion. Echocardiography is also a useful screening study for evaluating possible pericardial constriction, which can initially present with hepatic failure due to venous congestion.^25^ In addition, several systemic and infiltrative disorders, such as amyloidosis, hemochromatosis, and secondary iron overload, can coexist among patients with heart failure and liver disease. In such cases, advanced techniques, including echocardiographic strain imaging and cardiac magnetic resonance imaging, may be useful to establish diagnosis. The diagnosis of infiltrative systemic disorders is useful for OLT candidacy, both as it relates to long-term success of recipient organ and patient cardiac prognosis (ie, poorer prognosis in patients with progressive or advanced cardiac amyloidosis).

### RHC

Performing an RHC via the brachial vein is an attractive option because it may be performed independent of coagulopathy. Ultrasound guidance is a useful tool because it can help identify the brachial vein and facilitate vascular access. The right internal jugular vein is an alternative vascular access site as a large, superficial vessel that is easily compressible. Although femoral venous access is technically more challenging in patients who have large ascites or in obese patients with large pannus, it can be performed safely using ultrasound and fluoroscopic guidance.

Patients may proceed with OLT if the mean pulmonary artery pressure (PAP) is <35 mm Hg, pulmonary capillary wedge pressure (PCWP) is <15 mm Hg, and the pulmonary vascular resistance (PVR) is <3 Wood units (or <1200 dynes-sec/cm⁵). The diagnosis of pulmonary arterial hypertension (PAH) is made when the mean PAP is ≥20 mm Hg, PCWP is <15 mm Hg, and the PVR is >3 Wood units.^26^ These patients should be evaluated for pulmonary vasoreactivity with vasodilators including inhaled nitric oxide and intravenous epoprostenol and adenosine. Patients with pulmonary hypertension, and specifically PoPH, can demonstrate a positive response to vasoreactivity.^27^^,^^28^ Treatments using PAH-guided therapies to lower PAP have demonstrated a positive impact on mortality, as well as improving the candidacy and perioperative care following OLT. In observational studies, OLT may even be curative for pulmonary hypertension in select patients, obviating the need for long-term PAH-targeted therapies in such patients. A diagnosis of postcapillary pulmonary venous hypertension is made in patients with a mean PAP of ≥35 mm Hg, PCWP of >15 mm Hg, and PVR of <3 Wood units. These patients need diuretics but will likely be able to proceed with OLT with improvement in volume status and lowering of pulmonary artery and/or left heart pressures.

Portopulmonary hypertension occurs in 4% to 6% of patients with ESLD, is characterized by elevated mean PAP (≥20 mm Hg), low PCWP (≤15 mm Hg), and elevated PVR (≥3 Woods units) not explained by other causes of pulmonary hypertension, and merits a consultation with a pulmonary hypertension specialist.^29^, ^30^, ^31^, ^32^ Proposed mechanisms include an imbalance of vasoconstrictive and vasodilatory mediators bypassing liver metabolism, chronic microthromboembolism, genetic predisposition, hyperdynamic pulmonary circulation with pulmonary vascular remodeling, and inflammation.^33^^,^^34^ The natural history of PoPH leads to right heart failure, exercise limitation, and premature death. Before the availability of PAH-targeted therapies, studies reported a 1-year survival of 35% to 46%.^35^^,^^36^ The mortality rates of 36 nontreated patients with PoPH undergoing OLT were 100%, 50%, and 0% for those with a mean PAP of >50 mm Hg, >35 mm Hg but <50 mm Hg, and <35 mm Hg, respectively.^37^^,^^38^ For this reason, a mean PAP of >50 mm Hg should be considered a contraindication for OLT, whereas patients with a mean PAP of <35 mm Hg and normal right ventricular function may proceed. Treatment with PAH-targeted therapies and repeat RHC should be performed to assess response to therapy. A combination of PAH-targeted therapies and OLT resulted in excellent long-term survival in select patients with PoPH (81% at the 5-year follow-up).^39^ Data on the outcomes of combined lung-liver transplantation in patients with PoPH are limited.^40^ More data on dual organ transplant are needed to recommend this approach for OLT candidates with PoPH.

## Evaluation and treatment of CAD

It was previously postulated that liver disease may be protective against the development of coronary atherosclerosis because of impaired hepatic production resulting in improved lipid profiles, reduced clot formation, and low systemic vascular resistance with lower rates of hypertension. However, in several recent studies, the incidence and prevalence of CAD among patients with ESLD is similar, if not higher, compared with that of the general population.^5^^,^^41^, ^42^, ^43^ At least 1 critical coronary artery lesion (≥70% stenosis) was present in 5% to 26% of preoperatively asymptomatic OLT candidates.

Identification of hemodynamically significant CAD may be difficult among patients with ESLD, as symptoms of dyspnea may be attributed to volume overload rather than interpreted as angina. Several systematic reviews have been published examining the different modalities used to assess for CAD in OLT candidates.^44^, ^45^, ^46^ No consensus exists regarding the preferred diagnostic modality to exclude obstructive CAD in this specific population and the age cutoff for assessment for CAD, given the lack of evidence. Young patients (men aged <30 years and women aged <40 years) who are asymptomatic and have a normal electrocardiogram and TTE and are otherwise low-risk in the absence of risk factors (ie, nondiabetic, nonsmoker, and no evidence of familial hypercholesterolemia) are unlikely to benefit from testing for myocardial ischemia. As such, the authors do not recommend routine testing for myocardial ischemia in this low-risk cohort. Noninvasive physiologic or anatomic assessment is a reasonable option for patients with intermediate risk, including those with diabetes, prior cardiovascular disease, left ventricular hypertrophy, age of >60 years, smoking, hypertension, and dyslipidemia. Exercise stress testing should be considered, when possible. However, pharmacologic stress testing is often required because OLT candidates may not be able to complete exercise stress test protocols and reach the target heart rate for various reasons, including deconditioning and ascites.

### Myocardial perfusion imaging

Single-photon emission computed tomography (SPECT) has low sensitivity (37%) and specificity (61%) in OLT candidates^47^^,^^48^ (Table 1).^49^, ^50^, ^51^, ^52^, ^53^, ^54^ Patients with ESLD are commonly in a vasodilated state with impaired coronary flow reserve, resulting in coronary microvascular dysfunction and a decrease in coronary flow reserve, which may mitigate the effects of pharmacologic agents such as regadenoson and dipyridamole. In a study of 2500 patients, OLT candidates had abnormal perfusion defect in 7.8%, compared with 34.3% in non-OLT candidates.^55^ Of the 64 patients with abnormal SPECT who underwent subsequent invasive or noninvasive coronary angiography, obstructive CAD was observed in 25 (1.0%) patients, nonobstructive CAD was observed in 23 (0.9%) patients, and normal coronaries were found in 16 (0.6%) patients. Coronary revascularization was performed in 18 (0.7%) patients. A study of 339 patients with ESLD reported a negative predictive value of 99% in a low-risk cohort.^56^ Positron emission tomography (PET) is emerging as a potential strategy to improve the sensitivity relative to SPECT, although this has not been demonstrated specifically in OLT candidates. PET has been validated against dobutamine stress echocardiography, demonstrating concordance and may show further promise as further studies emerge.^45^^,^^57^

**Table 1:** Comparison of diagnostic tests used for evaluation of myocardial ischemia in general population

Reference	Modality	Sensitivity, %	Specificity, %	NPV, %	PPV, %	Radiation dose, mSv	Estimated test cost, $
Einstein,^49^ 2018 American Society of Nuclear Cardiology^50^	Tc-99m SPECT	35-100	50-88	77-100	15-30	∼9 to 12	1132
Mark et al,^51^ 2016	Stress echocardiography	9-32	78-98	75-89	2-37	0	514
Kosmala et al,^52^ 2019 Deseive et al,^53^ 2015	CCTA	95-99	83-91	83-99	64	∼3 to 5	404
MDsave^54^	Coronary angiography	n/a	n/a	n/a	n/a	∼2 to 5	9162

CCTA, coronary computed tomography angiography; n/a, not applicable; NPV, negative predictive value; PPV, positive predictive value; Tc-99m SPECT, technetium-99m single-photon emission computed tomography.

### Stress echocardiography

Dobutamine stress echocardiography in OLT candidates has poor sensitivity (13%-37%), high specificity (85%), and intermediate negative predictive value (75%) for obstructive CAD.^58^, ^59^, ^60^ This may be explained by a high cardiac output state, greater degrees of vasodilatation, likely because of higher levels of nitric oxide, impaired chronotropy, and autonomic dysfunction.^61^ Data on treadmill echocardiography in OLT candidates are lacking. Most OLT candidates are deconditioned and unable to exercise and reach the target heart rate.

### Coronary computed tomography angiography

Coronary computed tomography angiography (CCTA) is an attractive imaging modality in OLT candidates, especially in those who have coagulopathy, which obviates the need for transfusion of blood products if coronary angiography is considered. The sensitivity of CCTA, especially when it is performed using noninvasive fractional flow reserve (FFR), is high (87.9%).^62^ It is cost-effective and should also be considered in patients who are critically ill in the intensive care unit, many of whom are intubated and not candidates for noninvasive stress testing or may have increased risk with invasive procedures. Patients with chronic kidney disease should be adequately prehydrated to minimize the risk of contrast-induced nephropathy. Patients in normal sinus rhythm with a heart rate of <70 beats/min are good candidates, whereas patients who are tachycardic or have atrial fibrillation are not ideal candidates. Patients with left ventricular systolic dysfunction (LVEF of ≤40%), angina, or stress testing that shows mild ischemia or equivocal results could undergo confirmatory CCTA. The presence of severe coronary artery calcification or prior stents may preclude accurate assessment for the presence of obstructive CAD. In 1 study, the use of CCTA in OLT candidates confirmed the diagnosis of CAD in most cases, which obviated the need for coronary angiography.^63^ Up to 87.5% of coronary angiography would have been avoided if CCTA was performed in lieu of initial routine coronary angiography. No patient experienced a coronary event with this strategy. The authors concluded that an initial strategy of CCTA and reserving coronary angiography for inconclusive results or if revascularization was necessary may be useful for the evaluation of OLT candidates. Although CCTA is attractive for its high sensitivity and negative predictive value, computed tomography-fractional flow reserve (CT-FFR) for intermediate lesions has not been validated in patients with ESLD yet and may have limitations given the patient-specific assumptions in flow dynamics, assumptions of hyperemia, and microvascular function used in CT-FFR. Further studies are required to validate CT-FFR in patients with ESLD.

### Coronary angiography

The role of routine coronary angiography as the initial diagnostic modality is controversial. There is significant variation across different OLT centers. Some institutions perform coronary angiography in all OLT candidates, whereas other institutions reserve coronary angiography based on an age criterion (eg, aged ≥65 years) or other cardiac risk factors such as diabetes mellitus, hyperlipidemia, and tobacco use. However, many OLT candidates have coagulopathy and thrombocytopenia and are at increased risk of femoral artery vascular complications, including access site pseudoaneurysms (5.7%), major bleeding (14.8%), and need for blood products (16%), compared with matched controls.^64^^,^^65^ Reasonable values to proceed with coronary angiography are an international normalized ratio of <1.8 and platelet count of ≥50,000/μL, especially if vascular access will be obtained via the femoral artery. However, the preferred vascular access is the radial artery to minimize the risk of vascular access site complications. Routine coronary angiography may increase the likelihood of inappropriate and unnecessary coronary revascularization and utilization of valuable resources such as fresh frozen plasma in patients with elevated prothrombin time, platelets for thrombocytopenia, stents, and antiplatelet therapy.^66^

Indications for coronary angiography include the presence of high-risk features on noninvasive stress test (≥10% of myocardium at risk), evidence of obstructive CAD on CCTA (≥50% stenosis), symptomatic patients, and evaluation of patients with a history of revascularization for CAD or known obstructive CAD.

### Treatment of CAD

The decision to revascularize asymptomatic patients with ESLD with severe CAD is controversial. No prospective, randomized trial has shown that preoperative coronary revascularization improves clinical outcomes.^67^ Coronary revascularization should be based on clinical judgment and should be individualized with a multidisciplinary approach that includes the heart team (cardiologist and a cardiac surgeon) and OLT team. The risks and indications for percutaneous coronary intervention (PCI) must be weighed against the risks of delaying OLT due to completing dual antiplatelet therapy, especially in those with a high Model for End-Stage Liver Disease score, those who have respiratory failure requiring mechanical ventilation, or those with vasodilatory shock requiring vasopressor therapy. Patients who should be considered for revascularization include those with left main CAD and multivessel CAD involving the proximal-to-middle left anterior descending artery, especially those with left ventricular systolic dysfunction. Clinical appropriateness guidelines developed for coronary revascularization in patients with stable CAD have not been validated in OLT candidates, and functional criteria are often difficult to apply given the high rates of deconditioning in this patient cohort. Several studies have reported acceptable outcomes with coronary revascularization before OLT.^68^^,^^69^ In patients with severe multivessel CAD not amenable to PCI, coronary artery bypass graft surgery is often prohibitive because of high morbidity and mortality, especially in patients with ESLD with Child class B and C.^70^^,^^71^ One option to mitigate the high risk in these patients is to perform coronary artery bypass surgery at the time of OLT.^68^ If PCI is to be performed, drug-eluting stents with a treatment duration of 1 month with dual antiplatelet therapy appear to be safe among patients with a high bleeding risk and/or those with medical conditions that preclude long-term dual antiplatelet therapy.^72^^,^^73^ For patients with coagulopathy and thrombocytopenia who have high-risk coronary anatomy (left main disease or severe proximal left anterior descending artery) but are also at high bleeding risk and need PCI to become eligible for OLT, a short “trial” of dual antiplatelet therapy can be given for 1 to 2 weeks to ensure the ability to tolerate antiplatelet agent before performing PCI. Patients with high Model for End-Stage Liver Disease scores in the intensive care unit who require urgent OLT may not be able to wait the full 1 month of dual antiplatelet therapy. The use of cangrelor instead of clopidogrel as a perioperative bridge can be considered on a case-by-case basis. However, more data are needed to recommend this strategy.

Invasive assessment of intermediate stenoses using FFR or instantaneous wave-free ratio (iFR) are understudied and not validated within patients with ESLD. However, quantitative analysis of coronary flow reserve using Doppler echocardiography, magnetic resonance imaging, or PET have all demonstrated a reduction in flow reserve among patients with liver disease.^74^, ^75^, ^76^, ^77^ Similarly, it is possible that FFR may be inaccurate among patients with ESLD because of a baseline high coronary blood flow and an inability to induce hyperemia in an already maximally dilated microvasculature. From a practical standpoint, iFR is expected to have less interaction from microvascular function and would be expected to maintain accuracy in patients with ESLD, although future studies should seek to confirm its validation. At the present time, there are insufficient data on the accuracy of FFR or iFR to recommend its routine use to guide PCI in patients with ESLD. Intravascular imaging with intravascular ultrasound (IVUS) or optical coherence tomography can provide anatomic assessment of the coronary arteries and is not dependent on flow reserve. In the Fractional Flow Reserve and Intravascular Ultrasound-Guided Intervention Strategy for Clinical Outcomes in Patients with Intermediate Stenosis trial, FFR-guided PCI was noninferior to IVUS-guided PCI in patients with intermediate stenosis with respect to the composite primary end point of death, myocardial infarction, or revascularization at 24 months.^78^ The IVUS criteria for PCI was a minimum lumen area of ≤3 mm^2^ or between 3 and 4 mm^2^ with a plaque burden of >70%. Decisions to intervene on intermediate lesions should be made on an individual basis, taking into consideration patient-level factors (location of disease, risk of bleeding, symptoms of CAD, and urgency of OLT), and in conjunction with the heart-liver team approach.

Aspirin should be continued in all patients treated with PCI unless it is contraindicated, most commonly because of active bleeding, thrombocytopenia (<10,000/μL), or a history of variceal bleeding. Patients with CAD should be treated with a statin, unless contraindicated. Child class C patients with a bilirubin level of >5 mg/dL are at a high risk of adverse reactions to statin. Consultation with hepatology before statin initiation is recommended for these patients. The value of a statin in patients with very-low low-density lipoprotein level (<40 mg/dL) is uncertain. Although a beta-blocker is commonly used to treat portal hypertension, its use may be limited by the high prevalence of bradycardia and hypotension in patients with ESLD. Controversy surrounds its use to decrease the risk of perioperative ischemic complications.

## Current landscape and future directions

Given the complex pathophysiological changes and coagulopathies underlying cirrhosis, the optimal preoperative protocol for patients undergoing OLT remains challenging. We have summarized the most commonly used modalities for screening pulmonary hypertension and CAD within this cohort. However, important gaps remain in the available literature. These gaps may delay the adoption of these recommendations because of anecdotal bias and local practice but are critical to the design of future studies.

First, flow reserve is an important concept used to diagnose CAD based on the ability to maximally dilate and augment coronary blood flow. Given the vasodilatory state, it is unknown whether patients with ESLD have preservation of flow reserve. An impaired flow reserve reduces the accuracy of SPECT imaging, CT-FFR, and invasive angiographic assessment of intermediate stenoses. Second, because these tests are being used to screen patients, the cost-effectiveness of each approach must be considered. Thus, future studies should seek to reexplore the cost-effectiveness of CCTA versus coronary angiography specifically in OLT candidates. Third, future studies should seek to define the role of revascularization in patients undergoing OLT. Patients with ESLD who undergo PCI are at a high risk of bleeding, as these patients may also have varices or other risk factors for life-threatening bleeds, which could necessitate early cessation of antiplatelet agents and increase the risk of stent thrombosis. In addition, select patients may require urgent listing for OLT, and the risk of delaying OLT candidacy because of PCI may outweigh the benefit of revascularization. Future studies should explore coronary anatomy subtypes (eg, left main disease, multivessel CAD, and proximal left anterior descending artery), which are prohibitive to OLT unless revascularization is performed.

## Conclusions

This consensus document was written to help guide the cardiology community that evaluates and manages OLT candidates as clinical practice and protocols vary from institution to institution. Stress testing remains important in the pre-OLT evaluation to diagnose CAD and to guide coronary revascularization. However, it has limitations because of low sensitivity and specificity in this patient population. In this era of attention to appropriate use, cost containment and minimizing unnecessary invasive procedures, an initial strategy of CCTA should be encouraged as an alternative to routine coronary angiography. The role of revascularization in asymptomatic patients remains to be defined and requires further study. A shared decision-making approach with a multidisciplinary team while incorporating individual clinical circumstances is the best practice. Randomized trials are critically needed in this field and are needed to establish the standard of care.
